# Consomic mouse strain selection based on effect size measurement, statistical significance testing and integrated behavioral z-scoring: focus on anxiety-related behavior and locomotion

**DOI:** 10.1186/s12863-016-0411-4

**Published:** 2016-06-29

**Authors:** M. Labots, M. C. Laarakker, F. Ohl, H. A. van Lith

**Affiliations:** Division of Animal Welfare & Laboratory Animal Science, Department of Animals in Science and Society, Faculty of Veterinary Medicine, Utrecht University, P.O. Box 80166, 3508 TD Utrecht, The Netherlands; Brain Center Rudolf Magnus, University Medical Center Utrecht, Utrecht, The Netherlands; Present Address: Boston Scientific Nederland B.V., Nieuwegein, The Netherlands

**Keywords:** Anxiety-related behavior, Cohen’s *d*, Consomic mouse strain, Effect size, Integrated z-score, Locomotion, Partial eta squared (*η*_*p*_^*2*^)

## Abstract

**Background:**

Selecting chromosome substitution strains (CSSs, also called consomic strains/lines) used in the search for quantitative trait loci (QTLs) consistently requires the identification of the respective phenotypic trait of interest and is simply based on a significant difference between a consomic and host strain. However, statistical significance as represented by *P* values does not necessarily predicate practical importance. We therefore propose a method that pays attention to both the statistical significance and the actual size of the observed effect. The present paper extends on this approach and describes in more detail the use of effect size measures (Cohen’s *d*, partial eta squared - *η*_*p*_^*2*^) together with the *P* value as statistical selection parameters for the chromosomal assignment of QTLs influencing anxiety-related behavior and locomotion in laboratory mice.

**Results:**

The effect size measures were based on integrated behavioral z-scoring and were calculated in three experiments: (A) a complete consomic male mouse panel with A/J as the donor strain and C57BL/6J as the host strain. This panel, including host and donor strains, was analyzed in the modified Hole Board (mHB). The consomic line with chromosome 19 from A/J (CSS-19A) was selected since it showed increased anxiety-related behavior, but similar locomotion compared to its host. (B) Following experiment A, female CSS-19A mice were compared with their C57BL/6J counterparts; however no significant differences and effect sizes close to zero were found. (C) A different consomic mouse strain (CSS-19PWD), with chromosome 19 from PWD/PhJ transferred on the genetic background of C57BL/6J, was compared with its host strain. Here, in contrast with CSS-19A, there was a decreased overall anxiety in CSS-19PWD compared to C57BL/6J males, but not locomotion.

**Conclusions:**

This new method shows an improved way to identify CSSs for QTL analysis for anxiety-related behavior using a combination of statistical significance testing and effect sizes. In addition, an intercross between CSS-19A and CSS-19PWD may be of interest for future studies on the genetic background of anxiety-related behavior.

**Electronic supplementary material:**

The online version of this article (doi:10.1186/s12863-016-0411-4) contains supplementary material, which is available to authorized users.

## Background

Chromosome substitution strains (CSSs, also referred to as consomic strains or lines) have been developed as a tool to identify chromosomes harboring quantitative trait loci (QTLs) for complex phenotypes, such as behavioral traits. CSSs are produced by transferring a single chromosome from a donor strain unto the genetic background of a host strain through generations of backcrossing (for review: [1]). The selection of chromosomes that contain at least one QTL is carried out through the relatively simple process of comparing the phenotypes of each consomic line with the host strain, *i.e.* identification of statistical significance for the phenotypic difference between the host and consomic strain. In order to determine the position of the QTL(s) on a particular chromosome, a relatively small segregating population between the relevant chromosome substitution strain and the host strain is made. Combining genomic with phenotypic data of this population and performing specific statistical analyses (so-called QTL analyses) can result in the identification of significant or suggestive QTL(s) on a specific chromosome. An alternative approach for the location of the QTL(s) on the substituted chromosome is determined via CSS-derived congenic strains [2]. CSSs provide a tool for a more efficient genetic mapping by reducing the genetic complexity in a defined way [3, 4].

Identification of QTL harboring chromosomes via consomic strain surveys are based on statistical significance. However, statistical significance as represented by *P* values does not necessarily predicate practical significance [5, 6]. Still, it is a common misconception that statistical significance does equate large and/or (pre-)clinically/biologically relevant effects. We argue therefore that behavioral geneticists should be equally as interested in the actual size of the observed effect (*e.g.* Cohen’s *d*, partial eta squared - *η*_*p*_^*2*^) as in statistical significance. The *P* value depends essentially on two things: the size of the effect and the size of the sample. If all CSSs have the same sample size there is a simple mathematical relationship between the *P* value and the effect size; assuming that the same statistical test is used to generate the *P* value. In this situation the selection based on *P* value or on effect size will lead to a similar outcome. However in many consomic strain surveys sample sizes for the CSSs (and host strain) are different (see *e.g.* the behavioral genetic analyses of the consomic strain panels that are available in the Mouse Phenome Database (MPD; [7])). Furthermore, in many consomic strain surveys the host versus consomic strain comparisons are not always performed with the same statistical test. For example it could be based on Student’s *t* test, the Welch-Satterthwaite test or the Wilcoxon-Mann–Whitney test [8]. Thus we believe that the selection of consomic mouse strains should depend on more than solely the *P* value, but should also include the effect size. In a recent paper the Cohen’s *d* was introduced as a statistical parameter for selection of a consomic line [9]. The present paper extends on this approach and describes in more detail the use of effect size measurement (Cohen’s *d* and *η*_*p*_^*2*^) in addition to significance testing as statistical selection parameters for the chromosomal assignment of QTLs influencing modified Hole Board (mHB) behavior in laboratory mice. In the present study sample size per CSS (and host strain) deviate from each other (see Methods, *Animals and housing*: CSS, *n* = 6 or *n* = 27 or *n* = 31; C57BL/6J, *n* = 27 or *n* = 33).

To demonstrate this approach data from three different experiments was used. The first data set stems from a consomic strain survey that has been performed and is already published by our group [8]. In this survey (hereafter referred to as: experiment A) the CSSs were derived from A/J (donor strain) and C57BL/6J (host strain) progenitors and only male mice were tested. C57BL/6J mice were characterized to show less anxiety-related behavior than A/J mice (see Laarakker et al. [10] for an overview). Since sex differences are common in animal models of anxiety [10, 11], we subsequently tested female mice from the selected CSS from experiment A (C57BL/6J-Chr 19^A/J^/NaJ, simplified to CSS-19A) and their counterparts from the host strain (hereafter referred to as: experiment B). QTLs for the identified behavioral trait on mouse chromosome 19 can be mapped by using a (reciprocal) F_2_ intercross between CSS-19A and the C57BL/6J host strain. Further, by combining data from multiple crosses it is possible to narrow down the murine anxiety QTL interval(s) on chromosome 19. For this purpose a (reciprocal) F_2_ intercross between CSS-19A and another consomic mouse strain can be produced. Therefore, CSS-19PWD (C57BL/6J-Chr 19^PWD/PhJ^/ForeJ) was chosen, since it differs from C57BL/6J in the duration of thigmotaxis in the open field (OF) [12]. Thus, we tested male CSS-19PWD and PWD/PhJ mice with their C57BL/6J host strain (hereafter referred to as: experiment C).

For all three experiments we used the mHB test since this set up allows for comprehensive analysis of mouse behavior [13–15]. In a previous article on the behavioral genetic analysis of a chromosome substitution strain panel we reduced the variety of mHB measures to a small set of the summary scores using a principal component analysis (PCA) [8]. Guilloux et al. [16] proposed the use of integrated z-scores, instead of a PCA, as a sensitive and reliable method to present behavioral results for mice phenotyping. Like a PCA this method also reduces the measures to a smaller number of behavioral variables (= composite variables [17]). In the present paper the calculated effect size measures (Cohen’s *d* and *η*_*p*_^*2*^ values) are based on integrated z-scores, demonstrating how the selection of consomic mouse strains can be based on effect size measurement, statistical significance testing and integrated z-scoring in relation to anxiety-related behavior and locomotion in the mHB test.

## Methods

### General

The present animal study is reported in accordance with the so-called ARRIVE guidelines [18].

### Animals and housing

In this paper three different experiments (labeled A, B and C) were carried out to demonstrate our consomic strain selection approach. Experiment A was performed using naïve male mice from the following inbred strains: A/J (the donor strain, *n* = 30), C57BL/6J (the host strain, *n* = 27), and the complete set of chromosome substitution strains between these parental strains (*n* = 6 per consomic line). The nomenclature of the consomic lines is: C57BL/6J-Chr #^A/J^/NaJ and will be simplified in this experiment to CSS-#A (# = mouse chromosome number/letter). Extra male mice (*n* = 21) of the CSS-19A strain were tested. Data from this experiment *i.e.* the results for 35 behavioral variables measured/calculated in the mHB have been previously published [8]. Here we will report the effect size measures (*i.e.* Cohen’s *d* and *η*_*p*_^*2*^ values), as well as the statistical significance, based on integrated z-scores for anxiety-related behavior and locomotion in the mHB.

In experiment B naïve female C57BL/6J (*n* = 27) and CSS-19A (*n* = 27) mice were tested in the mHB. Additionally, in experiment C naïve male C57BL/6J (*n* = 33; consisting of *n* = 27 from experiment A and *n* = 6 extra animals in this experiment), PWD/PhJ (*n* = 6) and C57BL/6J-Chr 19^PWD/PhJ^/ForeJ (simplified to CSS-19PWD; *n* = 31) mice were behaviorally tested in the mHB. All animals of the three experiments were purchased from The Jackson Laboratory (Bar Harbor, ME, USA). Charles River Nederland B.V. (Maastricht, The Netherlands) coordinated the shipping of the animals from The Jackson Laboratory to the Utrecht University.

The animals were 4–6 weeks of age at arrival and were habituated to the environment and experimenter for at least two weeks prior to behavioral testing. All animals were housed at the Central Laboratory Animal Research Facility of Utrecht University (location ‘Paviljoen’). Testing took place in the same room as where the animals were housed. Testing equipment had been installed in this room prior to arrival of the animals. The animal room was sound-attenuated. Relative humidity was kept at a constant level of approximately 50 ± 5 %, the ambient temperature was maintained at 21 ± 2 °C and the ventilation rate was 15–20 air changes per hour. During the habituation period, all mice were handled at least four times a week for a few minutes by the person (MCL) performing the behavioral tests. Handling included picking up the animal at the tail base, placing it on the hand or arm and restraining it by hand for a few seconds at random times of the day.

The male animals (experiments A and C) were housed individually and female animals (experiment B) were housed socially with three mice in one cage. The wiretopped Macrolon® Type II-L (prolonged) cages (size: 365 × 207 × 140 mm, floor area 530 cm^2^; Techniplast, Milan, Italy) were enriched, besides standard bedding material (Aspen chips: Abedd-Dominik Mayr KEG, Köflach, Austria), with a tissue (Kleenex® Facial Tissues: Kimberly-Clark Professional BV, Ede, The Netherlands), a cardboard shelter (Technilab-BMI BV, Someren, The Netherlands) and a small amount (less than a handful) of paper shreds (EnviroDri®: Tecnilab-BMI BV). Drinking water and food (standard mice chow, Rat and Mouse Breeder and Grower Expanded-RM(E): Special Diet Services, Essex, UK) were provided *ad libitum* and the animals were housed under a reversed light–dark schedule (white light: 7:00 PM–7:00 AM [local circadian time], maximal 150 lux; red light: 7:00 AM–7:00 PM [local circadian time], maximal 5 lux). To reduce stress in the laboratory animal facility, radio sound (SkyRadio®, 60 ± 3 dB) was provided 24 h a day. The type of music was mainly easy-listening pop-music. In addition there was conversational radio-sound, which may accustom the animals to the human voice.

### Rationale sample sizes consomic strain survey

Using the classical approach in identifying specific QTLs with the use of consomic mouse lines often result in the use of a large numbers of animals. In order to reduce the number of animals necessary for these behavioral genetic experiments we previously suggested a two-stage approach using a consomic strain survey [8, 19]. We proposed to start the behavioral tests with 27 host strain animals and 6 animals per consomic lines (according to Belknap [20] a 4.5:1, or 27:6 ratio is the most efficient for selecting chromosome substitution strains that contain a QTL). Subsequently, and only if evidence was found for a specific chromosome harboring a behavioral QTL (*P* value < suggestive threshold for the host versus consomic strain mice comparison), testing of extra animals (*n* = 21) of the appropriate consomic strain was considered sensible. Finally, after behavioral testing, the statistical analysis was repeated, but now with 27 animals for both the consomic and host strain. This result delivers four possible scenarios, ultimately providing either significant evidence or no such evidence for a QTL, see Table 1. The choice of the number of mice per CSS in the first stage depends on the narrow sense heritability (*h*^*2*^) of the behavioral phenotype. Laarakker et al. [8] demonstrated that for behavioral phenotypes with a *h*^*2*^ ranging from 0.12 to 0.28, *n* = 6 per CSS (and *n* = 27 for the host strain) is sufficient to select consomics in the first stage.

**Table 1:** Overview of the possibilities in the two-stage approach of a consomic strain survey

Host (*n* = 27) versus consomic line (*n* = 6)			Host (*n* = 27) versus consomic line (*n* = 27)	
No	Suggestive	Significant	No	Significant
-	X	-	X	-
-	X	-	-	X
-	-	X	X	-
-	-	X	-	X
X^a^	-	-	X	-
X^a^	-	-	-	X

^a^ Very often several behavioral variables are measured in one behavioral test. For some of these variables there is neither evidence for a QTL in the *n* = 27 (host strain) versus *n* = 6 (consomic line) comparison nor in the *n* = 27 (host strain) versus *n* = 27 (consomic line) comparison. However it is also possible, but not likely, that significant evidence for a QTL turns up

### Behavioral testing

The behavior of the animals (age at testing 6–10 weeks) was assessed using the mHB, which is a single-test paradigm that can measure multiple motivational systems and behavioral dimensions, such as anxiety-related behavior (including avoidance, risk assessment and arousal) and activity-related behavior [13]. The procedure of the behavioral testing has previously been described in detail [8, 10]. The animals are placed in the set-up and behavioral variables (see Table 2 for a list of the variables measured in the mHB and used in this paper) were scored for 5 min by a trained observer (MCL). All behavioral scoring took place between 10:00 AM and 2:00 PM (*i.e.* during the active phase of the animals) under red-light conditions and all behavioral tests were videotaped (for raw data storage). The behavioral variables were live-scored using the computer software Observer 4.1 (Noldus, Wageningen, The Netherlands). Between behavioral tests, feces were removed from the test set-up, urine was wiped up and the experimental compartment was cleaned with tap water and paper towels.

**Table 2:** List of behavioral variables measured in the mHB and used in this study

MOTIVATIONAL SYSTEM
Behavioral dimension
Variable
ANXIETY-RELATED BEHAVIOR
Avoidance *[z]* ^a^
Total number of board entries (freq)*[−z]*
Latency until first the board entry (s) *[z]*
Percentage of time on the board (%) *[−z]*
Risk assessment *[z]*
Total number of risk assessments (freq) *[z]*
Latency until the first risk assessment (s) *[−z]*
Arousal *[z]*
Total number of self-groomings (freq) *[z]*
Latency until first self-grooming (s) *[−z]*
Percentage of time self-grooming (%) *[z]*
Total number of defecations (freq) *[z]*
Latency until first bolus (s) *[−z]*
Total number of urinations (freq) *[z]*
Latency until first urination (s) *[−z]*
LOCOMOTION
Total number of line crossings (freq) *[z]*
Latency until first line crossing (s) *[−z]*

^a^ The directionality of the z-scores was adjusted so that increased score values reflected increased values for that behavioral dimension or motivational system: *[z]* = regular z-score, *[−z]* = adjusted z-score

### Statistical analyses

All statistical analyses were carried out according to Field [21] using an IBM® SPSS® Statistics for Windows (version 22.0) computer program (IBM Corp., Armonk, NY, USA) and paying attention to the assumptions that underlie the various statistical procedures. Two-sided, exact (*i.e.* for the non-parametric tests) probabilities were estimated throughout. In order to assess the behavioral performance over the different experiments, the data was transformed using integrated z-scoring as proposed by Guilloux et al. [16]. With the modification that when determining the z-scores, it was calculated how many standard deviations (SD) an observation is above or below the mean of the pooled data (*i.e.* using the mean and SD of all animals in one experiment grouped together instead of normalizing to a reference group). Z-scores were calculated for each individual behavioral variable. Although it is not common for discrete numerical data, the means and SD for ‘total number-variables’ were also calculated and the variables were treated as continuous data as suggested by Fagerland et al. [22]. The direction of the z-scores was adjusted in a way that increased z-scores reflected increased values for that behavioral dimension (see Table 2). These individual z-scores were subsequently added and divided by the number of variables in a behavioral dimension to reflect an overall z-score on that behavioral dimension. The z-score for the anxiety motivational system was calculated by taking the mean of the z-scores for the behavioral dimensions avoidance, risk assessment and arousal behavior. The Kolmogorov-Smirnov one-sample test was used to check Gaussianity of the integrated behavioral z-score and covariate (see below) data. This was done per strain and led to the conclusion that for some strains the integrated z-score variables and/or the residuals were not normally distributed. In order to use these variables in a parametric analysis, a bootstrap method was applied (see next paragraph).

Group means per strain (donor or consomic) of the z-scores for the motivational systems and the behavioral dimensions were statistically compared with the group mean counterparts of C57BL/6J of each experiment. It has been described that using ancillary variables as covariates in the statistical analysis increase statistical power [23]. Therefore the host versus donor or consomic strain comparisons were performed with analyses of covariance (ANCOVAs, with *‘strain’* as main effect); the ancillary variables *‘season’* and *‘time of the day’* served as covariates, because there was evidence that these two variables influence the outcome of behavioral phenotyping [24, 25]. For the ANCOVAs, homoscedasticity was tested with the Levene’s test, which is a powerful and robust test based on the *F* statistic. Since the variances were not always equal and/or the within-strain data (*i.e.* the z-score variables for anxiety, avoidance, arousal and locomotion) as well as the residuals were not always normally distributed a bootstrap procedure (10,000 samples) was applied to the ANCOVA [26]. Covariate and bootstrap adjusted means and SDs were computed for the z-score variables for anxiety, avoidance, risk assessment, arousal and locomotion. The power for the factor *‘strain’* in the ANCOVAs could be calculated by SPSS and was extracted from the output. In order to estimate the *h*^*2*^, an ANCOVA with *‘strain’* as main factor and variables *‘season’* and *‘time of the day’* as covariates was carried out for the integrated behavioral z-scores across all 21 consomic strains (*n* = 6) and the host strain (*n* = 27). The sum of squares between strains divided by the total sum of squares, gives an estimate of the *h*^*2*^ of these z-scores (in experiment A) [20].

Locomotion or activity-related behavior can have a large influence on anxiety-related behavior. In order to assure that the effect of locomotion on anxiety-related behavior is controlled for, an additional ANCOVA (with 10,000 sample bootstrap procedure) was executed with *‘strain’* as main effect and *‘locomotion’* as a covariate in addition to *‘time of the day’* and *‘season’*.

The female and male CSS-19A and C57BL/6J mice originate partly from different batches. To exclude a significant batch effect an ANCOVA with factors *‘strain’*, *‘gender’* and *‘batch’*, and covariates *‘time of the day’* and *‘season’* was performed.

A correction for multiple comparisons should be taken into account to reduce the probability of a Type I error. Since this paper proposes an alternative method to selecting CSS strains to the PCA as described in Laarakker et al. [8], should the corrected statistical testing thresholds used in that study be in line with the current study. The threshold used in Laarakker et al. [8] was based on an article by Belknap [20], where it was stated that when comparing a CSS with a host strain, a significance threshold of *P* < 0.004 is acceptable and advisable. Resulting in a significance threshold in this paper of *P* < 0.004 for comparisons between donor or consomic and host strains (experiments A & C), host and CSS-19A (*n* = 27) or CSS-19PWD (*n* = 31) (experiment B or C respectively), and a suggestive threshold of 0.004 ≤ *P* < 0.05.

Statistical significance is not the same as practical significance, for which effect size analyses are more important [27]. Effect sizes reported include the partial eta squared values (*η*_*p*_^*2*^) within the ANCOVAs (with a 10,000 sample bootstrap procedure), as well as the Cohen’s *d* values based on adjusted means and SDs. The Cohen’s *d* is calculated as the difference between the adjusted mean of the overall z-score of a comparison group (donor or consomic strain) and that of the reference group (in this case the C57BL/6J host strain in each experiment) divided by the adjusted pooled SD. On the basis of a review by Wahlsten [28] of many studies with a wide variety of phenotypes, guidelines are offered for absolute values of Cohen’s *d* (|*d*|)that correspond to what are generally regarded as small, medium, large and very large effects in mouse neurobehavioral genetic studies: small effect, |*d*| ≤ 0.5; medium/moderate effect, 0.5 < |*d*| < 1.0; large effect, 1.0 ≤ |*d*| < 1.5; very large effect, |*d*| ≥ 1.5. The following cutoffs for the *η*_*p*_^*2*^ effect size coefficients were used: small effect, *η*_*p*_^*2*^ ≤ 0.03; medium/moderate effect, 0.03 < *η*_*p*_^*2*^ < 0.10; large effect, 0.10 ≤ *η*_*p*_^*2*^ < 0.20; very large effect, *η*_*p*_^*2*^ ≥ 0.20. The cutoffs for Cohen’s *d* and *η*_*p*_^*2*^ are different from and, in case of Cohen’s *d*, somewhat larger than values assigned to the same descriptors in psychological research with humans [29].

We consider that in a consomic strain survey very large chromosomal effects (Cohen’s *d* ≥ 1.5 and *η*_*p*_^*2*^ ≥ 0.20) together with *P* < 0.004 are indicative for significant evidence for a chromosome harboring a QTL. There is no evidence for a chromosome harboring a QTL if Cohen’s *d* < 1.0 and/or *η*_*p*_^*2*^ < 0.10 and/or *P* ≥ 0.05. All other cutoff combinations will result in suggestive evidence. Similar criteria have been used for a significant, suggestive or no evidence for a meaningful difference between host and donor strain. An overview of the different cutoff combinations together with the evidence for a meaningful QTL or a meaningful difference between host and donor strain can be found in Table 3.

**Table 3:** Type of evidence based on the combination of Cohen’s *d*, *η*_*p*_^*2*^ and *P* values

Cohen’s *d*	Partial eta squared (*η*_*p*_^*2*^)	*P* value	Evidence for a meaningful QTL/meaningful difference between host and donor strain
|*d*| < 1.0	*η*_*p*_^*2*^ < 0.10	*P* ≥ 0.05	No
|*d*| < 1.0	*η*_*p*_^*2*^ < 0.10	0.004 ≤ *P* < 0.05 (*/†)^a^	No
|*d*| < 1.0	*η*_*p*_^*2*^ < 0.10	*P* < 0.004 (**/††)	No
|*d*| < 1.0	0.10 ≤ *η*_*p*_^*2*^ < 0.20 (+)^a^	*P* ≥ 0.05	No
|*d*| < 1.0	0.10 ≤ *η*_*p*_^*2*^ < 0.20 (+)	0.004 ≤ *P* < 0.05 (*/†)	No
|*d*| < 1.0	0.10 ≤ *η*_*p*_^*2*^ < 0.20 (+)	*P* < 0.004 (**/††)	No
|*d*| < 1.0	*η*_*p*_^*2*^ ≥ 0.20 (++)	*P* ≥ 0.05	No
|*d*| < 1.0	*η*_*p*_^*2*^ ≥ 0.20 (++)	0.004 ≤ *P* < 0.05 (*/†)	No
|*d*| < 1.0	*η*_*p*_^*2*^ ≥ 0.20 (++)	*P* < 0.004 (**/††)	No
1.0 ≤ |*d*| < 1.5 (#)^a^	*η*_*p*_^*2*^ < 0.10	*P* ≥ 0.05	No
1.0 ≤ |*d*| < 1.5 (#)	*η*_*p*_^*2*^ < 0.10	0.004 ≤ *P* < 0.05 (*/†)	No
1.0 ≤ |*d*| < 1.5 (#)	*η*_*p*_^*2*^ < 0.10	*P* < 0.004 (**/††)	No
1.0 ≤ |*d*| < 1.5 (#)	0.10 ≤ *η*_*p*_^*2*^ < 0.20 (+)	*P* ≥ 0.05	No
1.0 ≤ |*d*| < 1.5 (#)	0.10 ≤ *η*_*p*_^*2*^ < 0.20 (+)	0.004 ≤ *P* < 0.05 (*/†)	Suggestive
1.0 ≤ |*d*| < 1.5 (#)	0.10 ≤ *η*_*p*_^*2*^ < 0.20 (+)	*P* < 0.004 (**/††)	Suggestive
1.0 ≤ |*d*| < 1.5 (#)	*η*_*p*_^*2*^ ≥ 0.20 (++)	*P* ≥ 0.05	No
1.0 ≤ |*d*| < 1.5 (#)	*η*_*p*_^*2*^ ≥ 0.20 (++)	0.004 ≤ *P* < 0.05 (*/†)	Suggestive
1.0 ≤ |*d*| < 1.5 (#)	*η*_*p*_^*2*^ ≥ 0.20 (++)	*P* < 0.004 (**/††)	Suggestive
|*d*| ≥ 1.5 (##)	*η*_*p*_^*2*^ < 0.10	*P* ≥ 0.05	No
|*d*| ≥ 1.5 (##)	*η*_*p*_^*2*^ < 0.10	0.004 ≤ *P* < 0.05 (*/†)	No
|*d*| ≥ 1.5 (##)	*η*_*p*_^*2*^ < 0.10	*P* < 0.004 (**/††)	No
|*d*| ≥ 1.5 (##)	0.10 ≤ *η*_*p*_^*2*^ < 0.20 (+)	*P* ≥ 0.05	No
|*d*| ≥ 1.5 (##)	0.10 ≤ *η*_*p*_^*2*^ < 0.20 (+)	0.004 ≤ *P* < 0.05 (*/†)	Suggestive
|*d*| ≥ 1.5 (##)	0.10 ≤ *η*_*p*_^*2*^ < 0.20 (+)	*P* < 0.004 (**/††)	Suggestive
|*d*| ≥ 1.5 (##)	*η*_*p*_^*2*^ ≥ 0.20 (++)	*P* ≥ 0.05	No
|*d*| ≥ 1.5 (##)	*η*_*p*_^*2*^ ≥ 0.20 (++)	0.004 ≤ *P* < 0.05 (*/†)	Suggestive
|*d*| ≥ 1.5 (##)	*η*_*p*_^*2*^ ≥ 0.20 (++)	*P* < 0.004 (**/††)	Significant

^a^ The symbols in parentheses (#, ##, +, ++, *, **, †, or ††) are also used in Figs. 1, 2 and 3

In order to compare the 9 orthogonal factors computed in Laarakker et al. [8] and the integrated behavioral z-scores in this paper, Spearman’s coefficients of rank correlation (*R*_*S*_) were calculated and significance was assessed by a two-tailed test based on the *t* statistic. Calculating numerous correlations also increases the risk of a Type I error. To avoid this, the level of statistical significance of Spearman correlation coefficients were adjusted using the Dunn-Šidák method (α = 1 – [1 – 0.05]^1/45^ ≈ 0.001139; 45 = total number of correlations [9 factors x 5 z-score variables]). In all other cases (*i.e.* Kolmogorov-Smirnov one sample test and Levene’s test), the probability of a Type I error < 0.05 was taken as the criterion of statistical significance.

## Results

### Experiment A

There were several, statistically significant correlations between the calculated integrated behavioral z-scores and the orthogonal factors computed in Laarakker et al. [8] (see Table 4). The variables *‘total number of line crossings’* and *‘latency until the first line crossing’* loaded highly on factor 1 and as a consequence this factor shows the highest association with the z-score for locomotion. Factor 2 reflected mainly ‘*avoidance’* behavioral variables (total number, latency, percentage of time) and appeared to associate highly with the z-scores for avoidance and anxiety. On factors 3 and 6 the arousal variables *‘grooming’* (total number, latency and percentage of time) and *‘boli produced’* (total number and latency) load highly. Therefore the z-score for arousal associated significantly with these two factors. The *‘risk assessment’* variables (total number and latency) loaded highly on factor 7. This factor associated highly with the z-score for risk assessment, but also with the z-score for overall anxiety.

**Table 4:** Associations (Spearman’s *R*_*s*_) between orthogonal factors and z-scores based on mHB behavioral variables^a^

	*z-scores*				
*Orthogonal factors* ^b^	*Anxiety*	*Avoidance*	*Risk assessment*	*Arousal*	*Locomotion*
Factor 1 – DI/ME/LO^c^	−0.185	−0.057	***−0.286***	−0.063	***0.634***
Factor 2 – AV/UN/OT	***−0.678***	***−0.950***	***−0.305***	−0.089	***0.233***
Factor 3 – AR	−0.154	0.023	0.054	***−0.573***	***0.312***
Factor 4 – OT	0.321	0.015	***0.373***	***0.312***	−0.003
Factor 5 – DI	−0.195	−0.184	***−0.214***	−0.030	***0.262***
Factor 6 – AR	−0.097	−0.002	0.131	***−0.495***	0.121
Factor 7 – RI/UN	***0.409***	0.060	***0.670***	0.048	***−0.242***
Factor 8 – UN	0.000	0.027	−0.114	0.027	0.171
Factor 9 – DI/ME	−0.036	−0.075	0.010	0.006	0.050

^a^ Association based on 204 animals. Significant (*P* < 0.001139) (Spearman’s *R*_*s*_) are indicated in ***bolditalics***

^b^ Orthogonal factors were taken from Laarakker et al. [8]

^c^ *AV* avoidance, *RI* risk assessment, *UN* undirected exploration, *DI* directed exploration, *ME* memory, *LO* locomotion, *AR* arousal, *OT* other behavior

The *h*^*2*^ of each z-score variable was found to be within the expected range of behavioral phenotypes in mice [30]: overall anxiety, *h*^*2*^ = 0.262; avoidance, *h*^*2*^ = 0.150; risk assessment, *h*^*2*^ = 0.265; arousal, *h*^*2*^ = 0.262; locomotion, *h*^*2*^ = 0.407. These *h*^*2*^ are high enough to select consomics in the first stage of our two step consomic line survey (*i.e.* with a host versus donor strain ratio of 27:6).

**Fig. 1:**
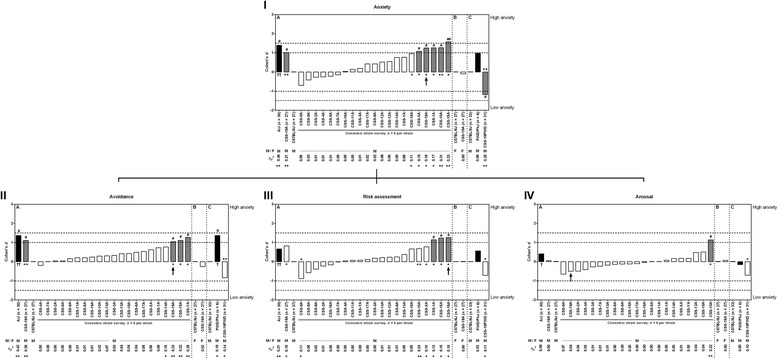
Effect sizes for (I) overall anxiety-related behavior, (II) avoidance, (III) risk assessment and (IV) arousal. The effect sizes (Cohen’s *d* and *η*_*p*_^*2*^) show the relative magnitude of the strain differences compared to C57BL/6J of each experiment. Inferential statistical comparison resulted in suggestive evidence * = 0.004 ≤ *P* < 0.05 for the consomic panel. For all other comparisons: ** = *P* < 0.004. (Chromosomal) effects are indicated as: # = *Large* (1.0 ≤ |*d*| < 1.5), ## = *Very Large* (|*d*| ≥ 1.5), + = *Large* (0.10 ≤ *η*_*p*_^*2*^ < 0.20) and ++ = *Very Large* (*η*_*p*_^*2*^ ≥ 0.20). M = males, F = females. Black bars: donor strains, white bars: CSS with no evidence for a meaningful QTL, chequered bars: CSS with suggestive evidence for a meaningful QTL

**Fig. 2:**
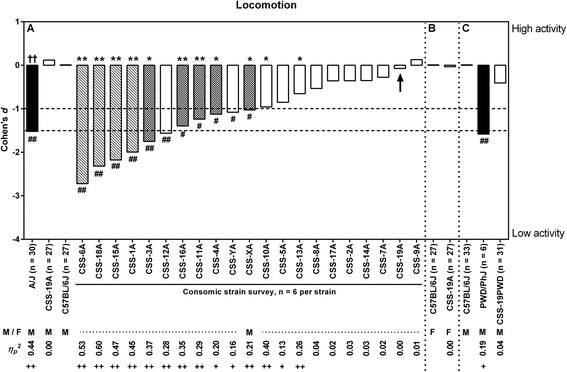
Effect sizes for activity-related behavior. The effect sizes (Cohen’s *d* and *η*_*p*_^*2*^) show the relative magnitude of the strain differences compared to C57BL/6J of each experiment Inferential statistical comparison resulted in suggestive evidence * = 0.004 ≤ *P* < 0.05 for the consomic panel. For all other comparisons: ** = *P* < 0.004. (Chromosomal) effects are indicated as: # = *Large* (1.0 ≤ |*d*| < 1.5), ## = *Very Large* (|*d*| ≥ 1.5), + = *Large* (0.10 ≤ *η*_*p*_^*2*^ < 0.20) and ++ = *Very Large* (*η*_*p*_^*2*^ ≥ 0.20). M = males, F = females. Black bars: donor strains, white bars: CSS with no evidence for a meaningful QTL, chequered bars: CSS with suggestive evidence for a meaningful QTL, bars with diagonal pattern: CSS with significant evidence for a meaningful QTL

For each strain (consomic and donor) and for every motivational system/behavioral dimension Cohen’s *d* and *η*_*p*_^*2*^ effect size coefficients were calculated (Figs. 1 and 2). All groups were compared to the C57BL/6J animals, since this is the host strain of the consomic lines, making the Cohen’s *d* and *η*_*p*_^*2*^ coefficients a measurement of the difference between the strains. The effect size of the donor strain (A/J) was *large/very large* (*i.e. d* ≥ 1.0 and *η*_*p*_^*2*^ ≥ 0.20) in avoidance behavior, *medium*/*very large* (*i.e.* 0.5 < *d* < 1.0 and *η*_*p*_^*2*^ ≥ 0.20) in risk assessment and *large*/*very large* in the overall anxiety score. The Cohen’s *d* for locomotion was negative and the measures for effect sizes were *very large*, meaning that A/J male mice show more anxiety-related behavior, but less locomotion compared to C57BL/6J. These findings are confirmed in the comparison of means of the behavioral dimensions: anxiety-related behavior, *F*_*1,53*_ = 45.683, bootstrap *P* < 0.000100; avoidance, *F*_*1,53*_ = 42.613, bootstrap *P* < 0.000100; risk assessment *F*_*1,53*_ = 15.970, bootstrap *P* = 0.000500; locomotion, *F*_*1,53*_ = 41.892, bootstrap *P* < 0.001800 (see also *P* values in Additional file 1: Table S1).

Comparing the panel of consomic lines with C57BL/6J regarding overall anxiety resulted in five lines with *large*/*verylarge* effect size and suggestive/significant *P* value (CSS-1A, CSS-10A, CSS-15A, CSS-19A and CSS-XA). All five lines showed suggestive evidence for a QTL for overall anxiety-related behavior (see Fig. 1-I). In the separate anxiety-related behavior dimensions, CSS-15A, CSS-19A and CSS-YA showed a *large*/*very large* effect size and suggestive *P* value in avoidance behavior (Fig. 1-II). CSS-1A, CSS-15A and CSS-19A showed a *large* effect size and suggestive *P* value for risk assessment and CSS-10A (*large*/*very large* effect size and suggestive *P* value) for arousal (see Fig. 1-III and IV). Finally, when considering the locomotion, almost all consomic lines showed a lower activity compared to C57BL/6J and only one consomic line showed a higher, albeit small and non-significant activity (CSS-9A; Fig. 2). CSS-19A (*n* = 6), on the other hand, showed almost no difference in locomotion compared to the control group (*d* = −0.07, *η*_*p*_^*2*^ = 0.0004, *F*_*1,29*_ = 0.013, bootstrap *P* = 0.908307). Considering this CSS-19A line showed a higher anxiety-related phenotype (in overall anxiety and separately in avoidance and risk assessment), but no difference in locomotion, was this line selected to be used in a QTL analysis for anxiety-related behavior. This consomic line was supplemented with 21 extra animals [8]. When supplemented to *n* = 27, the CSS-19A line stood out from the C57BL/6J regarding effect size and inferential statistical comparison (see also Additional file 1: Table S1) in overall anxiety-related behavior (*large*/*very large* effect size: *d* = 1.01, *η*_*p*_^*2*^ = 0.21; *F*_*1,50*_ = 13.584, bootstrap *P* = 0.000700) and avoidance behavior (*large*/*very large* effect size: *d* = 1.11, *η*_*p*_^*2*^ = 0.26; *F*_*1,50*_ = 17.362, bootstrap *P* = 0.000300), however not in risk assessment (*medium*/*large* effect size: *d* = 0.82, *η*_*p*_^*2*^ = 0.15; *F*_*1,50*_ = 9.015, bootstrap *P* = 0.004300), arousal (*small* effect size: *d* = 0.05, *η*_*p*_^*2*^ = 0.00; *F*_*1,50*_ = 0.024, bootstrap *P* = 0.874713) and locomotion (*small* effect size: *d* = 0.12, *η*_*p*_^*2*^ = 0.00; *F*_*1,50*_ = 0.200, bootstrap *P* = 0.648835).

**Fig. 3:**
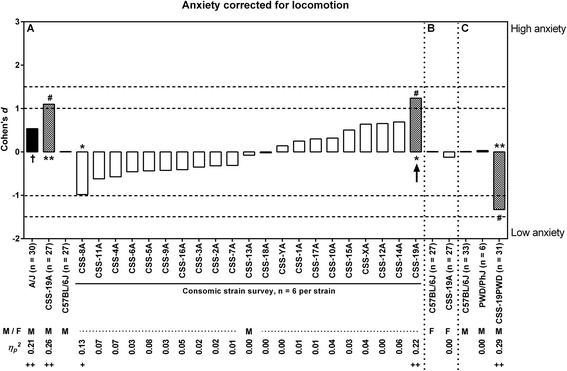
Effect sizes for anxiety-related behavior corrected for locomotion. The effect sizes (Cohen’s *d* and *η*_*p*_^*2*^), calculated from the values adjusted for locomotion, show the relative magnitude of the strain differences compared to C57BL/6J of each experiment Inferential statistical comparison resulted in suggestive evidence * = 0.004 ≤ *P* < 0.05 for the consomic panel. For all other comparisons: ** = *P* < 0.004. (Chromosomal) effects are indicated as: # = *Large* (1.0 ≤ |*d*| < 1.5), ## = *Very Large* (|*d*| ≥ 1.5), + = *Large* (0.10 ≤ *η*_*p*_^*2*^ < 0.20) and ++ = *Very Large* (*η*_*p*_^*2*^ ≥ 0.20). M = males, F = females. Black bars: donor strains, white bars: CSS with no evidence for a meaningful QTL, chequered bars: CSS with suggestive evidence for a meaningful QTL

Taking the possible effect of locomotion on anxiety-related behavior into account, the overall z-score for locomotion was incorporated as a covariate in an ANCOVA besides *‘time of day’* and *‘season’*. New effect sizes were calculated and statistical analysis was performed on the adjusted values originating from the ANCOVA (see Fig. 3). After the incorporation of the covariate *‘locomotion’*, only one strain (CSS-19A) showed suggestive evidence for a QTL with a *large*/*very large* effect size (*d* = 1.24, *η*_*p*_^*2*^ = 0.22; *F*_*1,28*_ = 8.044, bootstrap *P* = 0.000300). When supplemented to *n* = 27, the CSS-19A line showed compared to the host strain *large*/*very large* effect size (*d* = 1.10, *η*_*p*_^*2*^ = 0.26) and significant *P* value (*F*_*1,49*_ = 17.498, bootstrap *P* = 0.000300).

### Experiment B

Since male CSS-19A mice showed a higher anxiety-related phenotype compared to C57BL/6J (see experiment A), female CSS-19A and C57BL/6J were tested in the mHB. Strikingly, in overall anxiety (*small* effect size: *d* = −0.10, *η*_*p*_^*2*^ = 0.00; *F*_*1,50*_ = 0.142, bootstrap *P* = 0.723828), avoidance (*small* effect size: *d* = −0.25, *η*_*p*_^*2*^ = 0.02; *F*_*1,50*_ = 0.955, bootstrap *P* = 0.354665), risk assessment (*small* effect size: *d* = 0.02, *η*_*p*_^*2*^ = 0.00; *F*_*1,50*_ = 0.008, bootstrap *P* = 0.938806), arousal (*small* effect size: *d* = 0.07, *η*_*p*_^*2*^ = 0.00; *F*_*1,50*_ = 0.059, bootstrap *P* = 0.825717) and locomotion (*small* effect size: *d* = −0.03, *η*_*p*_^*2*^ = 0.00; *F*_*1,50*_ = 0.111, bootstrap *P* = 0.919808) CSS-19A showed similar behavior compared to C57BL/6J female mice (Figs. 1 and 2). The incorporation of *‘locomotion’* as a covariate for anxiety-related behavior resulted in a *small* effect (*d* = −0.12, *η*_*p*_^*2*^ = 0.00*, F*_*1,49*_ = 0.142, bootstrap *P* = 0.690831) (Fig. 3).

### Experiment C

CSS-19PWD showed less anxiety-related behavior compared to the host strain (anxiety: *large*/*very large* effect size *d* = −1.18, *η*_*p*_^*2*^ = 0.25, *F*_*1,60*_ = 19.580, bootstrap *P* = 0.000100; avoidance: *medium*/*large* effect size *d* = −0.83, *η*_*p*_^*2*^ = 0.14, *F*_*1,60*_ = 9.690, bootstrap *P* = 0.002300; risk assessment: *medium*/*large* effect size *d* = −0.72, *η*_*p*_^*2*^ = 0.11, *F*_*1,60*_ = 6.730, bootstrap *P* = 0.011499; arousal: *medium* effect size *d* = −0.71, *η*_*p*_^*2*^ = 0.10, *F*_*1,60*_ = 6.073, bootstrap *P* = 0.006799) (Fig. 1). Also, CSS-19PWD exhibited an activity comparable to the host strain (*small*/*medium* effect size *d* = −0.41, *η*_*p*_^*2*^ = 0.04, *F*_*1,60*_ = 2.371, bootstrap *P* = 0.134487) (Fig. 2). There was suggestive evidence for a meaningful difference between PWD/PhJ and C57BL/6J for avoidance behavior, but not for overall anxiety-related behavior, risk assessment and arousal (anxiety: *medium* effect size *d* = 0.98, *η*_*p*_^*2*^ = 0.09, *F*_*1,35*_ = 3.474, bootstrap *P* = 0.164898; avoidance: *large* effect size *d* = 1.37, *η*_*p*_^*2*^ = 0.18, *F*_*1,35*_ = 7.863, bootstrap *P* = 0.008111; risk assessment: *small*/*medium* effect size *d* = 0.56, *η*_*p*_^*2*^ = 0.03, *F*_*1,35*_ = 0.145, bootstrap *P* = 0.416700; arousal: *small* effect size *d* = −0.16, *η*_*p*_^*2*^ = 0.00, *F*_*1,35*_ = 0.079, bootstrap *P* = 0.782139) (Fig. 1). The PWD/PhJ showed, contrastingly, a higher avoidance phenotype compared to the host strain (Fig. 1) and a large effect in locomotion (*large* effect size *d* = −1.58, *η*_*p*_^*2*^ = 0.19, *F*_*1,35*_ = 6.073, bootstrap *P* = 0.223848), where PWD/PhJ showed less activity (Fig. 2). Finally, the incorporation of the covariate *‘locomotion’* resulted in the following effects on anxiety-related behavior in CSS-19PWD and PWD/PhJ: *d* = −1.32, *η*_*p*_^*2*^ = 0.29, *F*_*1,59*_ = 19.580, bootstrap *P* = 0.000100 and *d* = 0.03, *η*_*p*_^*2*^ = 0.00, *F*_*1,34*_ = 3.474, bootstrap *P* = 0.943123 respectively (Fig. 3).

### Power calculation

**Fig. 4:**
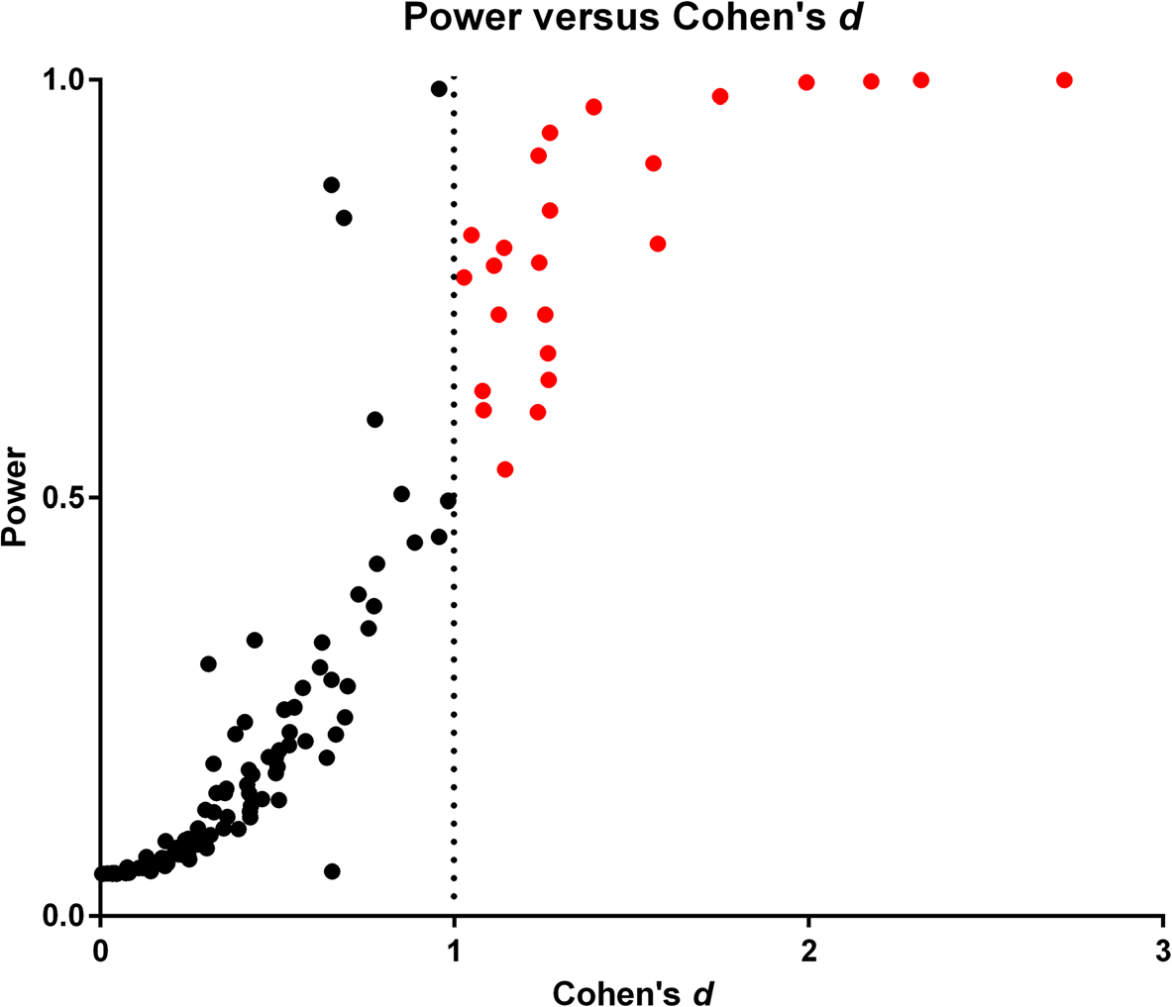
Scatterplot of calculated power and Cohen’s *d*. Based on *n* = 126 and the ratio host strain: consomic line = 27:6. The consomic line versus host strain comparisons with a relevant effect (*i.e.* |*d*| ≥ 1.0) are indicated in red. The average power of the comparisons with |*d*| ≥ 1.0 is 80.8 %

For the various host versus consomic strain comparisons in the first stage of the consomic strain survey from experiment A, the power for the factor *‘strain’* was calculated. These values were plotted against the Cohen’s *d* (Fig. 4). The average power of the *large*/*very large* effect sizes (|*d*| ≥ 1.0) was 80.8 % (indicated in red in Fig. 4).

## Discussion

Here we propose an alternative method to use effect sizes (Cohen’s *d* and *η*_*p*_^*2*^) in combination with statistical significance testing (*P* value) in the selection of a suitable consomic mouse strain using integrated behavioral z-scoring. The use of integrated behavioral z-scoring reduces the behavioral variables measured to a motivational system/behavioral dimension describing in this case, anxiety- and activity-related behavior comparable to a PCA. As expected there were statistically significant associations between the calculated integrated behavioral z-scores and the computed orthogonal factors from the PCA performed in Laarakker et al. [8] (Table 4). Although a negative association resulted from the correlation study between Factor 2 and the z-scores for anxiety, avoidance and risk assessment, this does not necessarily mean that there is a negative association between the anxiety-related behavior measured for Factor 2 and the z-scores, since the PCA factors were Varimax rotated [8] in order to fit a structure similar to other variables which may facilitate interpretation [31]. The z-scores for the behavioral dimensions were then used to calculate the effect sizes in order to estimate the magnitude of the strain difference between a specific CSS strain and the host strain (here C57BL/6J). Conventionally, strain differences are measured in an inferential statistical comparison and are expressed in *P* values. However, solely looking at *P* values can leave out a distinction between a large or a small effect [32]. In contrast, using measures for effect size (in addition to the *P* value) show the magnitude of the difference between two strains. A comparison of the different analysis methods from a previous study [8] and the present study, and the outcome of the consomic panel survey using different variables for anxiety-related behavior is shown in Table 5. In our proposed approach, where an overall z-score was calculated for anxiety-related behavior, chromosomes 1, 10, 15, 19 and X were indicated to possibly possess one or multiple QTLs (Fig. 1-I). However, using this approach and selecting CSSs that do possess anxiety-related (Fig. 1-I) but not locomotion QTL(s) (Fig. 2), only CSS-19A meets the criterion. Following this procedure in our previous study [8] we may select CSS-8A, CSS-17A, CSS-19A and CSS-YA.

**Table 5:** Suggestive and significant evidence for QTLs influencing anxiety-related behavior and/or locomotion in the mHB of male inbred mice^a^

*MOTIVATIONAL SYSTEM / Behavioral dimension /* Behavioral variables	Chromosomes																					Type of analysis^b^
	1	2	3	4	5	6	7	8	9	10	11	12	13	14	15	16	17	18	19	X	Y	
	*QTLs influencing the difference in mHB behavior between male C57BL/6J and A/J mice*																					
*Locomotion*	**X** ^c^	–	x	x	–	**X**	–	–	–	–	x	–	–	–	**X**	–	–	**X**	–	x	–	Z
Total number of line crossings	**X**	–	**X**	**X**	x	**X**	–	–	–	**X**	**X**	**X**	**X**	–	**X**	**X**	–	**X**	–	x	–	U
Latency until the first line crossing	x	–	–	–	–	**X**	–	–	x	x	–	x	–	–	x	–	–	**X**	–	–	–	U
Factor 1 – DI/ME/LO^d^	**X**	–	x	–	–	**X**	x	–	–	**X**	x	x	x	–	–	–	–	**X**	–	–	–	U
*ANXIETY* corrected for *Locomotion*	–	–	–	–	–	–	–	–	–	–	–	–	–	–	–	–	–	–	x	–	–	Z
*ANXIETY*	x	–	–	–	–	–	–	–	–	x	–	–	–	–	x	–	–	–	x	x	–	Z
*Avoidance*	–	–	–	–	–	–	–	–	–	–	–	–	–	–	x	–	–	–	x	–	x	Z
Total number of board entries	–	–	–	–	–	–	–	–	–	–	–	–	–	–	–	–	–	–	**X**	–	–	U
Latency until the first board entry	–	–	–	–	x	–	–	–	–	–	–	–	–	–	–	–	–	–	**X**	–	x	U
Percentage of time on the board	–	–	–	–	–	–	–	–	–	–	–	–	–	–	–	–	–	–	–	–	–	U
Average duration of a board entry	–	–	–	–	–	–	–	x	–	**X**	–	–	–	–	–	–	–	–	–	–	–	U
Latency until the first board entry + Percentage of time on the board	–	–	–	–	–	–	–	–	–	–	–	–	–	–	x	–	–	–	–	–	**X**	B
Latency until the first board entry + Average duration of a board entry	–	–	–	–	–	–	–	–	–	x	–	–	–	–	**X**	–	–	x	x	–	**X**	B
Factor 2 – AV/UN/OT	–	–	–	–	–	–	–	–	–	–	–	–	–	–	–	–	–	–	–	–	x	F
*Risk assessment*	x	–	–	–	–	–	–	–	–	–	–	–	–	–	x	–	–	–	x	–	–	Z
Total number of stretched attends	x	–	–	–	–	–	–	–	–	**X**	–	–	–	–	**X**	–	–	x	**X**	x	–	U
Latency until the first stretched attend	–	–	–	–	–	–	–	–	–	x	–	–	–	–	–	–	–	–	**X**	x	–	U
Factor 7 – RI/UN	**X**	–	–	–	–	x	–	–	–	**X**	–	x	–	–	**X**	–	–	x	x	**X**	–	F
*Arousal*	–	–	–	–	–	–	–	–	–	x	–	–	–	–	–	–	–	–	–	–	–	Z
Total number of self-groomings	–	–	–	–	–	–	–	–	–	–	–	–	–	–	–	–	–	–	–	–	–	U
Latency until the first self-grooming	–	–	–	–	–	x	–	–	–	–	–	–	–	–	–	–	–	–	–	–	–	U
Percentage of time self-grooming	–	–	–	–	–	–	–	–	–	–	–	–	–	–	–	–	–	–	–	–	–	U
Average duration of a self-grooming	–	–	–	–	–	–	–	–	–	–	–	–	–	–	–	–	–	–	–	–	–	U
Latency until the first self-grooming + Percentage of time self-grooming	–	–	–	–	–	–	–	–	–	–	–	–	–	–	–	–	–	–	–	–	–	B
Latency until the first self-grooming + Average duration of a self-grooming	–	–	–	–	–	–	–	–	–	–	–	–	–	–	–	–	–	–	–	–	–	B
Total number of defecations	–	–	–	–	–	–	–	–	–	x	–	–	–	–	–	–	–	–	–	–	–	U
Latency until the first bolus	–	–	–	–	–	–	–	–	–	–	–	–	–	–	–	–	–	–	–	–	–	U
Total number of urinations	–	–	–	–	–	–	–	–	–	x	–	–	–	–	–	–	–	–	–	–	–	U
Latency until the first urination	–	–	–	–	–	–	–	–	–	x	–	–	–	–	–	–	–	–	–	–	–	U
Factor 3 – AR	–	–	–	–	–	**X**	–	–	–	–	–	–	–	–	–	–	–	–	–	–	–	U
Factor 4 – OT	–	–	x	–	x	–	–	–	–	–	–	–	–	–	–	–	x	–	–	–	**X**	F
Factor 6 – AR	–	–	–	–	–	–	–	–	–	–	–	–	–	–	–	–	–	–	–	–	–	F

^a^ Based on *n* = 27 host and *n* = 6 consomic mice

^b^ Finding evidence for a QTL on a particular chromosome was based on different methods: Z = effect size measurement, statistical significance testing (ANCOVA plus bootstrapped *P* values) and integrated behavioral z-scoring [this study]; U = univariate statistical analysis [8] (unpaired Student’s *t* test, unpaired Student’s *t* test with Welch-Satterthwaite correction, Wilcoxon-Mann–Whitney test); B = bivariate statistical analysis [8] (Hotelling’s *T* ^*2*^ test); F = Factor analysis [8]

^c^ **X** = significant, x = suggestive, and – = no evidence for a QTL on a particular chromosome

^d^ The factors are labelled with the behavioral dimensions they mainly reflect. *Abbreviations used: AR* arousal, *AV* avoidance, *DI* directed exploration, *LO* locomotion, *ME* memory, *OT* other behavior, *RI* risk assessment, *UN* undirected exploration

Previous studies reported a phenotype for A/J that was characterized by relatively high anxiety-related behavior as compared to C57BL/6J in unconditioned behavior tests (*e.g.* Trullas & Skolnick [33], Van Gaalen & Steckler [34], Bouwknecht & Paylor [35], Wahlsten & Crabbe [36], Pletcher [37], Schalkwyk et al. [38], Laarakker et al. [10], Wiltshire & Pletcher [39], Molenhuis et al. [40]). However, other studies showed opposing results with *e.g.* A/J mice revealing less avoidance behavior in the open field (Brown et al. [41], Donahue et al. [42]), EPM (Brown et al. [41]) and elevated zero maze (Brown et al. [41]) as compared to C57BL/6J animals. The presently proposed method showed significantly (*P* < 0.004) higher anxiety-related behavior for the A/J mouse strain as compared to C57BL/6J animals (with Cohen’s *d* ≥ 1.0 and *η*_*p*_^*2*^ ≥ 0.20). Comparing A/J with C57BL/6J mice, Laarakker et al. [10] showed this higher anxiety-related phenotype for A/J when testing animals in the mHB, where Bouwknecht & Paylor [35] found similar findings in the light–dark transitions test as well as Van Gaalen & Steckler [34] that studied a number of inbred mouse strains in a series of anxiety-related tests. A number of studies in the Mouse Phenome Database (MPD) also reported higher anxiety-related behavior in A/J mice compared to C57BL/6J in a number of behavioral setups (Trullas & Skolnick [33], Wahlsten & Crabbe [36], Pletcher [37], Schalkwyk [38], Wiltshire & Pletcher [39]) and, finally, Molenhuis et al. [40] found higher anxiety-related behavior in A/J animals compared to C57BL/6J individuals when comparing 4 inbred strains in the elevated plus maze. For locomotion the A/J mice showed significantly (*P* < 0.004) less activity-related behavior than C57BL/6J (Cohen’s *d* ≤ −1.5 and *η*_*p*_^*2*^ ≥ 0.20) also confirming previous studies (*e.g.* Molenhuis et al. [40], Van Gaalen & Steckler [34] and Laarakker et al. [10]).

With an increasing interest from the research community for sex differences [43], the use of female test subjects and the fact that emotional disorders (including anxiety and depression) are more prevalent in females [44], experiment B was performed with females from the host strain C57BL/6J and CSS19-A. However, the effect sizes were close to zero for the anxiety-related behavioral dimensions/motivational system and locomotion; also, inferential statistical comparisons resulted in no significant or suggestive differences (Figs. 1 and 2). This is in accordance with earlier studies that have shown that males and females can respond differently or even in opposite directions when studying anxiety and depression (for reviews: Palanza [11] and ter Horst et al. [43]). For instance a study by Võikar et al. [45], reported either less avoidance behavior by the females or no difference when testing both males and females from multiple mouse lines in the light–dark box, open field and elevated plus maze. The female and male CSS-19A and C57BL/6J mice originate partly from different batches. Thus, the difference between the effects found in experiment A and the lack of effect in experiment B could be due to differences between batches. The effect of these different batches was controlled using an ANCOVA with main factors *strain*, *gender* and *batch* with covariates ‘time of day’ and ‘season’. No significant batch effects were detected, disproving any batch effects as a cause of the difference between experiments A and B.

**Fig. 5:**
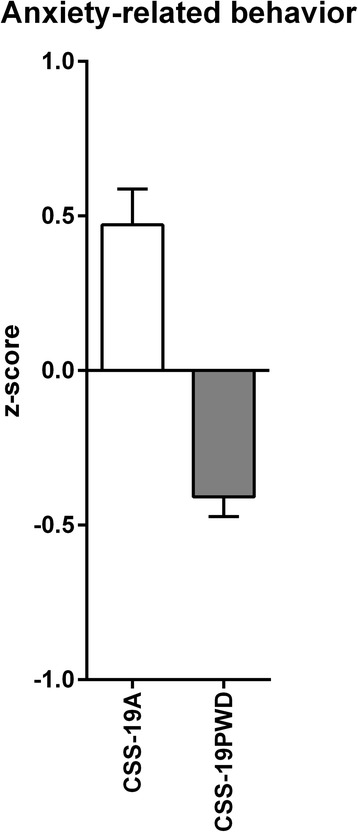
Mean anxiety-related behavioral z-scores for CSS-19A and CSS-19PWD. The bars represent mean +/− SEM of the overall anxiety-related z-score measured in the mHB and calculated from male *n* = 27 CSS-19A and *n* = 31 CSS-19PWD

**Fig. 6:**
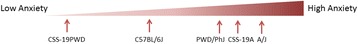
Spectrum of anxiety-related behavior for A/J, C57BL/6J, PWD/PhJ, CSS-19A and CSS-19PWD. The relative distance between the five mouse strains on a scale from low anxiety-related behavior to high anxiety-related behavior

Since evidence has been found for the presence of a QTL for anxiety-related behavior on chromosome 19 using male CSS-19A and C57BL/6J [8], the idea emerged to increase the variation between the parental strains by using a cross between CSS-19A and a consomic strain that was also found to exhibit different anxiety-related behavior compared to C57BL/6J. The consomic strain CSS19-PWD came into view, since the MPD shows the CSS-19PWD to exhibit a longer duration of thigmotaxis in the OF, a measure of anxiety-related behavior, compared to C57BL/6J [12]. In line with these findings CSS-19PWD also show similar activity-related behavior to C57BL/6J in the OF [12]. Surprisingly, in the mHB the opposite becomes evident for CSS-19PWD in the overall anxiety motivational system, showing a decreased anxiety-related phenotype compared to C57BL/6J (see Figs. 1-I, 5 and 6). A possible confounding procedural difference may underlie differences found between the OF and mHB results. Regarding general activity, there was no meaningful difference in locomotion between CSS-19PWD and C57BL/6J. Similar to the findings reported in the MPD for the OF test [12], the donor strain PWD/PhJ, as compared to C57BL/6J, is less active and exhibits more avoidance behavior in the mHB (suggestive evidence for a meaningful difference).

This study shows a large contrast (= suggestive evidence for a meaningful difference) between CSS19-PWD and C57BL/6J animals in terms of overall anxiety, but not locomotion. Therefore, an intercross between CSS19-A and CSS19-PWD (Cohen’s *d* = 1.81) may be of interest for future studies on the genetic background of anxiety-related behavior (see Figs. 5 and 6). A computer simulation with R/qtl [46] and R/qtlDesign [47] of the power and LOD score threshold for such linkage study is shown in Table 6, where different sample sizes, the assumption that one or two QTL’s would be present on chromosome 19, the position of the QTL(s), the amount of genotypic errors or missing data, and the composition of the genetic map were used. In the case of two QTLs present on chromosome 19, a power of >80 % would be established with a sample size of 99 animals. This is 12 % more than the ~80 progeny suggested by Singer et al. [48].

**Table 6:** Estimated power and LOD score threshold based on 10,000 simulation replicates for F2 intercross between CSS-19A and CSS-19PWD^a^

Number of male F_2_ animals (LOD threshold)^b^	Genotype data^c^	Position of additive QTL(s): at length of MMU19 (≈ cM)			Power	
		¼ (≈14.6)	½ (≈29.2)	¾ (≈43.7)	Markers (24) equally spaced	Randomly positioned markers (24)
					Average spacing = 2.5 cM	Average spacing = 2.5 cM
					Maximum spacing = 2.5 cM	Maximum spacing = 7.5 cM
*N* = 33	Error-free / complete	+	–	–	0.5489	0.5593
(2.4520)	1 % G / 5 % M	+	–	–	0.5613	0.5574
	Error-free / complete	–	+	–	0.5528	0.5657
	1 % G / 5 % M	–	+	–	0.5695	0.5422
	Error-free /complete	–	–	+	0.5453	0.5585
	1 % G / 5 % M	–	–	+	0.5663	0.5438
	Error-free /complete	+	+	–	0.3129	0.3227
	1 % G / 5 % M	+	+	–	0.3363	0.3142
	Error-free / complete	+	–	+	0.3027	0.2993
	1 % G / 5 % M	+	–	+	0.3087	0.3003
	Error-free / complete	–	+	+	0.3296	0.3303
	1 % G / 5 % M	–	+	+	0.3454	0.3078
*N* = 66	Error-free / complete	+	–	–	0.9122	0.9102
(2.3497)	1 % G / 5 % M	+	–	–	0.9118	0.8958
	Error-free / complete	–	+	–	0.9078	0.9100
	1 % G / 5 % M	–	+	–	0.9128	0.8838
	Error-free / complete	–	–	+	0.9086	0.9128
	1 % G / 5 % M	–	–	+	0.9152	0.8920
	Error-free / complete	+	+	–	0.6511	0.6513
	1 % G / 5 % M	+	+	–	0.6556	0.6097
	Error-free / complete	+	–	+	0.5973	0.5823
	1 % G / 5 % M	+	–	+	0.6076	0.5513
	Error-free / complete	–	+	+	0.6516	0.6410
	1 % G / 5 % M	–	+	+	0.6599	0.6025
*N* = 99	Error-free / complete	+	–	–	0.9885	0.9891
(2.3321)	1 % G / 5 % M	+	–	–	0.9898	0.9874
	Error-free / complete	–	+	–	0.9871	0.9867
	1 % G / 5 % M	–	+	–	0.9878	0.9867
	Error-free / complete	–	–	+	0.9898	0.9873
	1 % G / 5 % M	–	–	+	0.9887	0.9892
	Error-free / complete	+	+	–	0.8551	0.8351
	1 % G / 5 % M	+	+	–	0.8511	0.8434
	Error-free / complete	+	–	+	0.7865	0.7878
	1 % G / 5 % M	+	–	+	0.7958	0.7938
	Error-free / complete	–	+	+	0.8427	0.8402
	1 % G / 5 % M	–	+	+	0.8564	0.8456
*N* = 132	Error-free / complete	+	–	–	0.9989	0.9987
(2.3086)	1 % G / 5 % M	+	–	–	0.9990	0.9983
	Error-free / complete	–	+	–	0.9988	0.9988
	1 % G / 5 % M	–	+	–	0.9990	0.9986
	Error-free / complete	–	–	+	0.9993	0.9982
	1 % G / 5 % M	–	–	+	0.9990	0.9993
	Error-free / complete	+	+	–	0.9466	0.9413
	1 % G / 5 % M	+	+	–	0.9447	0.9446
	Error-free / complete	+	–	+	0.9118	0.9057
	1 % G / 5 % M	+	–	+	0.9110	0.9069
	Error-free	–	+	+	0.9427	0.9423
	1 % G / 5 % M	–	+	+	0.9479	0.9399

^a^ The genetic length of male mouse chromosome 19 is 58.309 cM [49]

^b^ Estimate of the 5 % LOD threshold

^c^ ‘1 % G / 5 % M’ means 1 % genotyping errors and 5 % missing data

## Conclusions

The proposed method shows a more extensive way to select a consomic mouse strain for QTL analysis. When searching for a QTL for anxiety-related behavior the following three points should be considered: i) effect sizes: |*d*| ≥ 1.0 and *η*_*p*_^*2*^ ≥ 0.1; ii) *P* < 0.05; iii) Is there evidence for an anxiety-related behavioral QTL, but not for a locomotion QTL? Based on these criteria the consomic strain survey (host strain, C57BL/6J; donor strain, A/J) indicated that only mouse chromosome 19 likely contains at least one anxiety-related behavioral QTL. For future genetic studies it is of interest to set up an intercross between CSS-19A and CSS-19PWD, because these two consomic lines differ markedly in anxiety-related behavior (Cohen’s *d* = 1.81) without a pleiotropic contribution of locomotion.

## Abbreviations

ANCOVA, Analysis of covariance/analysis with variance with covariate(s); AR, arousal; AV, avoidance; CSS-#A (# = mouse chromosome number/letter), C57BL/6J-Chr #^A/J^/NaJ; CSS, chromosome substitution strain; CSS-19PWD, C57BL/6J-Chr 19^PWD/PhJ^/ForeJ; *d*, Cohen’s *d* effect size; DI, directed exploration; *h*^*2*^, narrow sense heritability; LO, locomotion; LOD, Logarithm of the odds (base-10 log likelihood ratio); ME, memory; mHB, modified hole board; MPD, Mouse Phenome Database; OF, open field; OT, other behavior; PCA, principal component analysis; QTL, quantitative trait locus; RI, risk assessment; *R*_*S*_, Spearman’s coefficient of rank correlation; SD, standard deviation; SEM, standard error of the mean; UN, undirected exploration; *η*_*p*_^*2*^, partial eta squared effect size

## Additional file

Additional file 1: Table S1. Bootstrap *P* values of comparison of means between C57BL/6J and donor or consomic lines in mHB behavioral dimensions and motivational systems. (DOCX 20 kb)
