# Supplementary material for: Consomic mouse strain selection based on effect size measurement, statistical significance testing and integrated behavioral z-scoring: focus on anxiety-related behavior and locomotion
Source: BMC Genet. 2016 Jun 29;17:95. doi: 10.1186/s12863-016-0411-4 (PMC4928255; doi:10.1186/s12863-016-0411-4)
Supplement: Additional file 1: Table S1. — Bootstrap P values of comparison of means between C57BL/6J and donor or consomic lines in mHB behavioral dimensions and motivational systems. (DOCX 20 kb) [file 12863_2016_411_MOESM1_ESM.docx]

**Table S1: Bootstrap *P* values of comparison of means between C57BL/6J and donor or consomic lines in mHB behavioral dimensions and motivational systems.**

| **Experiment** |  | *P* values | | | |
| --- | --- | --- | --- | --- | --- |
| *(C57BL/6J versus)* | Z-Anxiety | Z-Avoidance | Z-Risk Assessment | Z-Arousal | Z-Locomotion |
| **Experiment A *(males)***  *(*0.004 ≤ *P* < 0.05 / *P < 0.004)^1^* | | |  |  |  |
| A/J *(n = 30)* | **0.000100^2^** | **0.000100** | **0.000500** | 0.038196 | **0.001800** |
| CSS-19A *(n = 27)* | **0.000700** | **0.000300** | 0.004300 | 0.874713 | 0.648835 |
| CSS-1A *(n = 6)* | 0.026479 | 0.236320 | 0.009930 | 0.871569 | **0.000100** |
| CSS-2A *(n = 6)* | 0.602806 | 0.947310 | 0.589780 | 0.300963 | 0.389880 |
| CSS-3A *(n = 6)* | 0.641625 | 0.430877 | 0.732204 | 0.601725 | 0.005013 |
| CSS-4A *(n = 6)* | 0.576447 | 0.713223 | 0.984914 | 0.355949 | 0.022629 |
| CSS-5A *(n = 6)* | 0.653399 | 0.654509 | 0.170492 | 0.955872 | 0.051318 |
| CSS-6A *(n = 6)* | 0.403368 | 0.375263 | 0.583400 | 0.788677 | **0.000100** |
| CSS-7A *(n = 6)* | 0.730827 | 0.966249 | 0.981062 | 0.571758 | 0.505663 |
| CSS-8A *(n = 6)* | 0.102515 | 0.968653 | 0.030865 | 0.788519 | 0.344188 |
| CSS-9A *(n = 6)* | 0.276225 | 0.792347 | 0.509122 | 0.170575 | 0.672848 |
| CSS-10A *(n = 6)* | **0.000701** | 0.511274 | **0.000903** | 0.019050 | 0.007314 |
| CSS-11A *(n = 6)* | 0.830828 | 0.707469 | 0.941165 | 0.991680 | **0.003209** |
| CSS-12A *(n = 6)* | 0.256264 | 0.410714 | 0.558186 | 0.304971 | 0.097625 |
| CSS-13A *(n = 6)* | 0.232041 | 0.241182 | 0.311323 | 0.579411 | 0.004610 |
| CSS-14A *(n = 6)* | 0.123297 | 0.082715 | 0.578805 | 0.739523 | 0.385671 |
| CSS-15A *(n = 6)* | **0.002004** | 0.030856 | 0.005112 | 0.810364 | **0.000501** |
| CSS-16A *(n = 6)* | 0.961905 | 0.629912 | 0.693695 | 0.929638 | **0.000301** |
| CSS-17A *(n = 6)* | 0.306397 | 0.361779 | 0.779525 | 0.831714 | 0.408120 |
| CSS-18A *(n = 6)* | 0.038720 | 0.394547 | 0.050576 | 0.349925 | **0.000100** |
| CSS-19A *(n = 6)* | 0.031676 | 0.046803 | 0.006508 | 0.326625 | 0.908307 |
| CSS-XA *(n = 6)* | 0.016029 | 0.236396 | 0.021746 | 0.698667 | 0.010028 |
| CSS-YA *(n = 6)* | 0.160201 | 0.034269 | 0.898397 | 0.747545 | 0.098727 |
| **Experiment B *(females)*** *(P<0.004)* |  |  |  |  |  |
| CSS-19A *(n = 27)* | 0.723828 | 0.354665 | 0.938806 | 0.825717 | 0.919808 |
| **Experiment C *(males)*** *(P<0.004)* |  |  |  |  |  |
| PWD/PHJ *(n = 6)* | 0.164898 | 0.008111 | 0.416700 | 0.782139 | 0.223848 |
| CSS-19PWD *(n = 31)* | **0.000100** | **0.002300** | 0.011499 | 0.006799 | 0.134487 |

**^1^** Threshold for significance in experiment A, B and C is *P* < 0.004. Significant comparisons indicated in ***bold italics.*** Suggestive evidence in study A is 0.004 ≤ *P* < 0.05, indicated in *underlined italics*.

^2^ *P* values obtained from bootstrapped ANCOVA with *strain* as main effect and ‘time of day’ and ‘season’ as covariates.
